# Synergistic Effect of Biosynthesized Silver Nanoparticles and Natural Phenolic Compounds against Drug-Resistant Fish Pathogens and Their Cytotoxicity: An In Vitro Study

**DOI:** 10.3390/md19010022

**Published:** 2021-01-08

**Authors:** Ehab Essawy, Mohamed S. Abdelfattah, Mansour El-Matbouli, Mona Saleh

**Affiliations:** 1 Clinical Division of Fish Medicine, University of Veterinary Medicine, 1210 Vienna, Austria; mansour.El-Matbouli@vetmeduni.ac.at (M.E.-M.); mona.saleh@vetmeduni.ac.at (M.S.); 2 Department of Chemistry, Faculty of Science, Helwan University, Cairo 11795, Egypt; mabdelfattah@science.helwan.edu.eg; 3 Helwan Nanotechnology Center, Helwan University, Cairo 11795, Egypt; 4 Marine Natural Products Unit, Faculty of Science, Helwan University, Cairo 11795, Egypt

**Keywords:** AgNPs, rutin, heliomycin, synergistic effect, fish pathogens, EPC

## Abstract

Fish pathogens causing disease outbreaks represent a major threat to aquaculture industry and food security. The aim of the presented study is to develop safe and effective bioactive agents against two bacterial isolates: *Aeromonas hydrophila* and *Pseudomonas fluorescens*. We employed a broth microdilution method to investigate the antibacterial effect of biosynthesized silver nanoparticles (AgNPs); rutin, a natural flavonoid extracted from *Ruta graveneoles*; and heliomycin, a secondary metabolite produced by marine actinomycetes AB5, as monotherapeutic agents. Moreover, AgNPs in combination with rutin (AgNP + R) and heliomycin (AgNPs + H) were examined for their synergistic effect. The cytotoxic effect of individual bioactive compounds and in combination with AgNPs was investigated on epithelioma papulosum cyprini (EPC) fish cell lines. Individual treatment of AgNPs, rutin, and heliomycin exhibited a dose-dependent antimicrobial activity against *A. hydrophila* and *P. fluorescens*. Rutin minimum inhibitory concentration (MIC) showed the lowest cytotoxicity when tested on EPC cell lines, while heliomycin MIC was highly cytotoxic. Combined subtherapeutic doses of AgNPs + R and AgNPs + H displayed additive and synergistic effects against *A. hydrophila* and *P. fluorescens*, respectively, with improved results and relative safety profile. The study findings demonstrate that a combination of AgNPs and natural bioactive compounds may represent novel therapeutics fighting fish pathogens potentially affecting the fish farming industry.

## 1. Introduction

Sustainment of healthy farmed fish is a significant economic and ecological capital in both developed and developing countries [1]. Fish pathogens causing disease outbreaks are considered a major threat, alarming the aquaculture industry and food security [2] especially after the emergence of several antibiotic-resistant bacterial strains [3]. *Aeromonas hydrophila* is a fish pathogen that causes hemorrhagic septicemia syndrome [4], leading to increased mortality in aquaculture [5]. The conventional antibiotic treatment against *A. hydrophila* is limited because of its ability to form a biofilm with diversified exopolysaccharide (EPS) [6]. *Psedomonas fluorescens* is a Gram-negative bacterium existing in diverse ecological niches. It infects a variety of farmed fish, including grass carp (*Ctenopharyngodon idella*), tilapia (*Oreochromis* spp.), trout, and Japanese flounder (*Paralichthys olivaceus*) [5]. Fish infected with *P. fluorescens* develop “red skin” disease that can lead to heavy mortality [7]. Hence, investigations for new therapeutics against *A. hydrophila* and *P. fluroscens* are required to control the emergence of high virulent strains due to the misuse of antibiotics [8].

Recently, modern treatment modalities for fish diseases have been developed using bionanotechnology [9]. Metal nanoparticles (NPs) such as gold NPs, silver NPs (AgNPs), and zinc oxide NPs showed antimicrobial activities against several fish pathogens, enabling an alternative approach for controlling disease outbreaks [10,11]. In our previous study, AgNPs exhibited antibacterial and antifungal activities including *Aeromonas salmonicida* and *Aphanomyces invadans*, respectively [12]. Moreover, we recommended chitosan nanoparticles to control invasive bacterial species [13]. However, safety concerns have been raised over the usage of AgNPs due to their potential side effects [14]. Oral administration of chemically synthesized AgNPs in *Labeu rohita* lead to bioaccumulation in gills, liver, and muscles. Moreover, a marked alteration in hematological parameters and pathological damage in tested tissues were observed [15].

**Figure 1:**
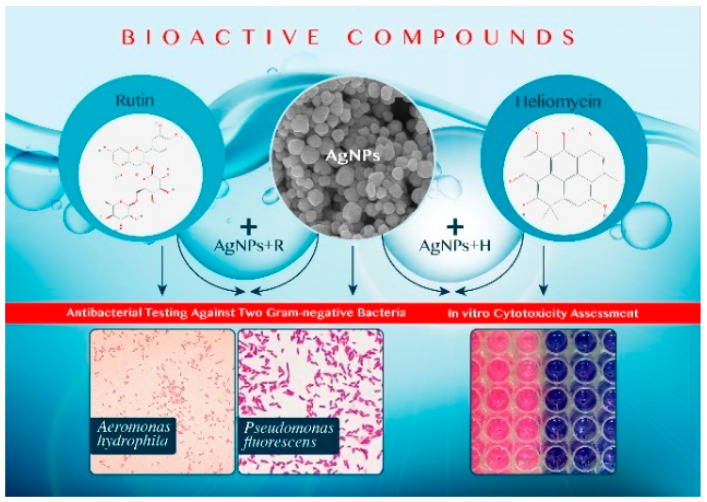
Graphical representation for testing the synergistic/additive effects of silver nanoparticles (AgNPs) to rutin and heliomycin against *Aeromonas hydrophila* and *Pseudomonas fluorescens*; and cytotoxcity testing.

Therefore, new bioactive compounds for safe and effective antimicrobial therapy should be considered. Biosynthesized AgNPs are supposed to have a better safety level [16]. Additionally, plant-derived flavonoids, a large group of naturally occurring phenylchromones found in fruits and vegetables, have diverse biological activities [17]. Rutin (3, 3′, 4′, 5, 7 pentahydroxyflavone-3-hamnoglucoside) is a flavonoid glycosidic compound found mainly in lemons, oranges, berries, limes, and grapes. Rutin exhibits a variety of biological and pharmacological activities, including antioxidant, antidiabetic, immunomodulatory, anti-inflammatory, and neuroprotective effects [18,19]. Heliomycin is a secondary metabolite released from marine actinomyctes. It is an aromatic polyketide in structure [20] and exhibits antibacterial, antiviral, and anticancer activities [21,22]. Heliomycin inhibits RNA polymerase [23] that leads to apoptosis [22] and inhibition of RNA and protein synthesis. Additionally, it acts as a histone deacetylase inhibitor [24]. Therefore, we postulated that combined treatment of natural products and biosynthesized AgNPs might offer a balanced solution between efficacy and safety. In the present study, we aimed to develop novel, safe, and effective therapeutic compounds against the two bacterial strains affecting the aquaculture industry. To achieve this goal, we tested biosynthesized AgNPs, rutin, and heliomycin, and combinations of AgNPs and rutin (AgNPs + R) and AgNPs and heliomycin (AgNPs + H) against *A. hydrophila* and *P. fluorescens* (Figure 1). A cytotoxicity study was performed to test the compounds on epithelioma papulosum cyprinid (EPC) fish cell lines to evaluate their safety. We believe that it is the first study on the synergistic inhibitory effects of biosynthesized AgNPs, phytogenic compounds, and secondary metabolites on the pathogens affecting aquaculture, namely *A. hydrophila* and *P. fluorescens.* Both Gram-negative strains were selected as they are potentially pathogenic for fish and humans, more cross resistant to popular antibiotics, and more abundant in aquaculture fish and water [25].

## 2. Results

### 2.1. Characterization of the Biosynthesized AgNPs (Particle Size and Zeta Potential)

**Figure 2:**
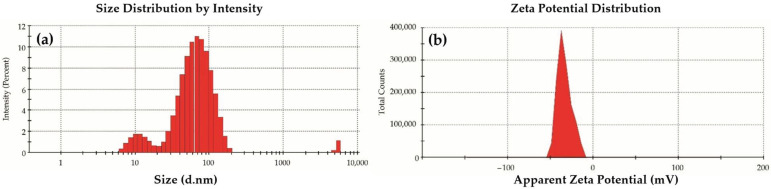
Characterization of biosynthesized AgNPs. (**a**) Particle size distribution with intensity peaks at 12.1 nm and 73.7 nm, average size of 56.1 nm, and polydispersity index (PDI) of 0.36, which indicates good homogeneity of the AgNPs. (**b**) Zeta potential distribution with maximum charge of −33.8. All data are expressed as means ± SD (*n* = 3).

The mean size and size distribution profile of the biosynthesized AgNPs are shown in Figure 2. The particle size distribution shows two intensity peaks at 12.1 nm and 73.7 nm, with an average diameter of 56.1 nm and a polydispersity index (PDI) of 0.36, which indicates good homogeneity of the AgNPs (Figure 2a). The surface potential of −33.8 mv (Figure 2b) indicates a good stability against coalescence.

### 2.2. Antibacterial Effects of Bioactive Compounds

#### 2.2.1. Minimum Inhibitory Concentration (MIC)

A considerable dose-dependent antibacterial effect of rutin, heliomycin, and AgNPs against both bacterial isolates *A. hydrophila* and *P. fluorescens* was observed by the broth microdilution method, which is evident from the explored MIC values (Table 1). The lowest MIC value was recorded for AgNPs (2 µg/mL) against *A. hydrophila*, followed by rutin against *P. fluorescens*, and the highest MIC value was recorded for heliomycin at 2048 µg/mL against *A. hydrophila*.

#### 2.2.2. Determining Minimum Bactericidal Concentration (MBC)

**Figure 3:**
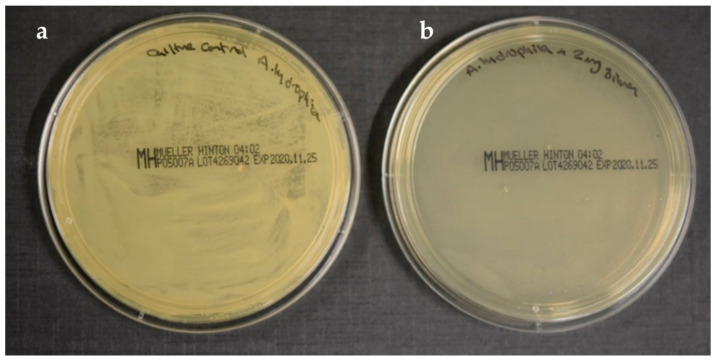
The bactericidal effect of silver nanoparticles against *A. hydrophila.* (**a**) Bacterial culture of *A. hydrophila*. (**b**) Treated plate with AgNPs (2 µg/mL).

The lowest treatment concentration that prevented 99.9% of the bacterial growth on agar plates was recorded at 20 µg/mL AgNPs against *A. hydrophila* (Figure 3). The bactericidal effect of rutin was observed for both tested strains and inhibited their colonial growth (Table 1). The lowest MBC value was recorded against *P. fluorescence* at 20 µg/mL of AgNPs, indicating high susceptibility, while the highest MBC value was recorded at 2048 µg/mL of rutin against *A. hydrophila*. Heliomycin exhibited bacteriostatic activity against both strains but could not inhibit their colonial growth at the tested concentrations (Table 1).

#### 2.2.3. Synergy Studies

A serial dilution of rutin and heliomycin sub-MIC—0.5 MIC, 0.25 MIC, 0.125 MIC, and 0.062 MIC—was selected for combinations with AgNPs sub-MIC of 1 µg/mL (Table 2, Table 3 and Table 4).

#### 2.2.4. Synergistic Effect Using the Disc Diffusion Method

**Figure 4:**
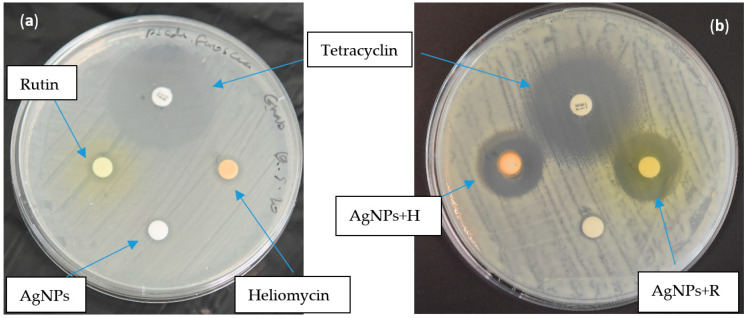
Disc diffusion assay results for the antibacterial effect of tested bioactive compounds against *P. fluorescens*. (**a**) Zone of inhibition for individual compounds: rutin R (512 µg/mL), heliomycin R (512 µg/mL), AgNPs (2 µg/mL), and tetracycline (10 µg) as positive control. (**b**) Widening of inhibition zone (arrow) for the combination of AgNPs + R and AgNPs + H at the same concentrations.

A wider zone of inhibition was observed (Figure 4) with the combination of 0.5 MIC of AgNPs (2 µg/mL) with 0.5 MIC of rutin and heliomycin (512 µg/mL), which indicates a synergistic effect of the combinations (Table 5).

#### 2.2.5. Effect of Bioactive Compounds on Bacterial Growth

**Figure 5:**
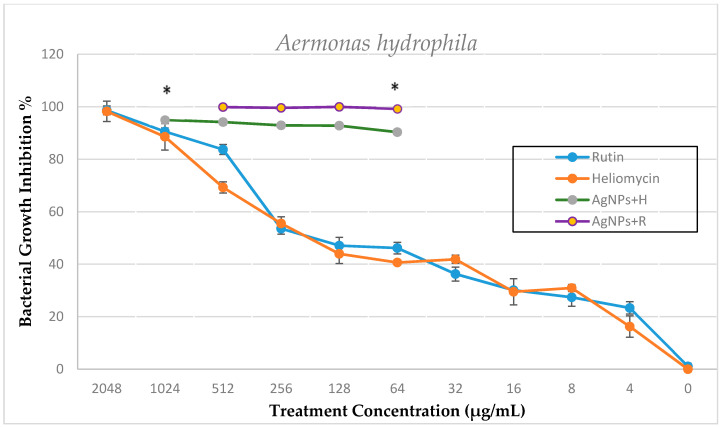
Percent inhibition of *A. hydrophila* growth when tested against a serial dilution of rutin and heliomycin (2048 µg/mL to 0 µg/mL) and sub-MIC combinations of AgNPs + rutin (R) and AgNPs+ heliomycin (H). All data are expressed as means ± SD (*n* = 3). Error bars represent the standard deviation from average values. * Statistical significance from control (*p* < 0.05).

**Figure 6:**
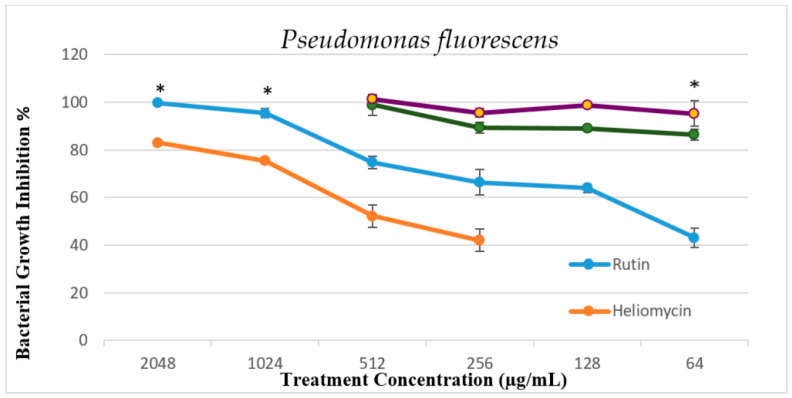
Percent inhibition of *P. fluorescens* growth when tested against serial dilutions of rutin and heliomycin (2048 µg/mL to 64 µg/mL) and sub-MIC combinations of AgNPs + rutin (R) and AgNPs + heliomycin (H). All data are expressed as means ± SD (*n* = 3). Error bars represent the standard deviation from average values. * Statistical significance from control (*p* < 0.05).

**Figure 7:**
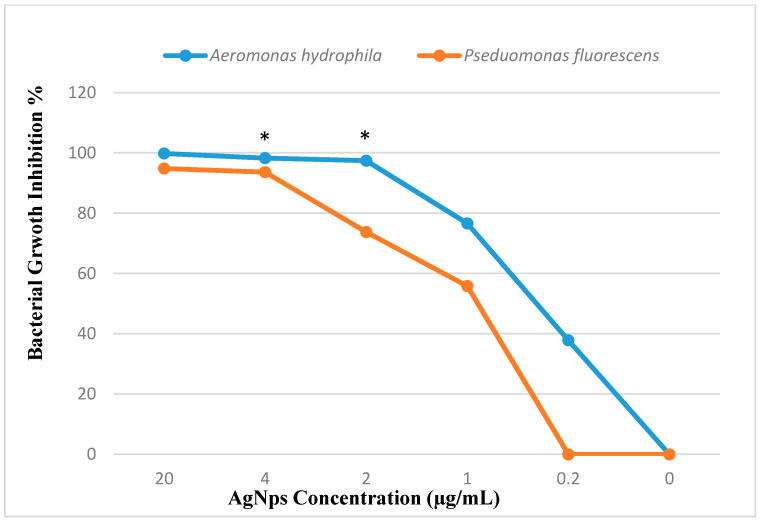
Percent inhibition of *A. hydrophila* and *P. fluorescens* at tested silver nanoparticles AgNPs doses (0–20 µg/mL). All data are expressed as means ± SD (*n* = 3). * Statistical significance from control (*p* < 0.05).

The bacterial growth inhibition of bioactive compounds alone and in combination with silver nanoparticles was confirmed by the broth microdilution method after overnight incubation at 22 °C by lower absorbance capacities at 600 nm using an absorbance microplate reader, Tecan Sunrise (Männdorf, Switzerland). The results show a dose-dependent antibacterial activity of the tested compounds and their bactericidal or bacteriostatic effect against *A. hydrophila* and *P. fluorescens*, as shown in Figure 5, Figure 6 and Figure 7.

### 2.3. Cytotoxicity Assessment of Bioactive Compounds

#### 2.3.1. Cell Morphology

**Figure 8:**
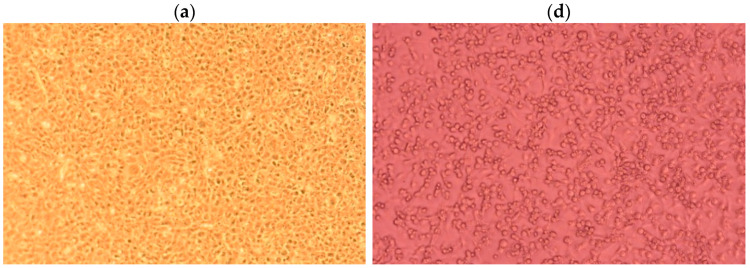

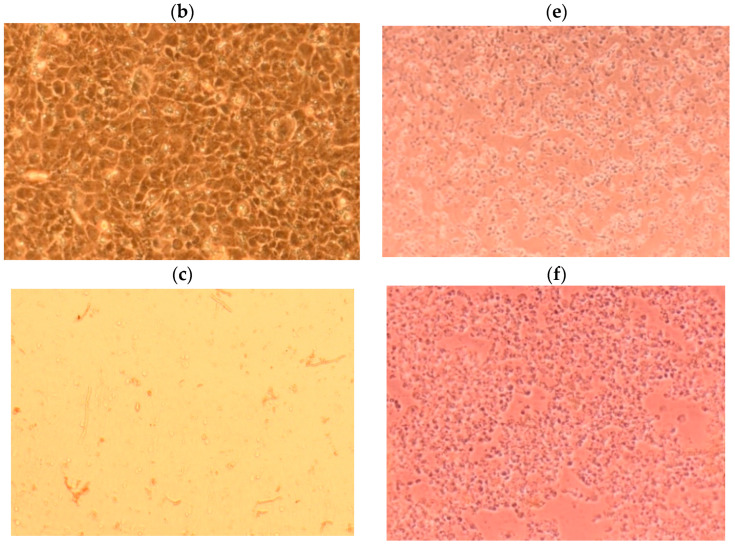
Morphological changes of epithelioma papulosum cyprinid (EPC) fish cell lines exposed to MIC of tested treatments for 24 h. No morphological changes were observed in control cells (**a**) and in rutin-treated cells at 1024 µg/mL, (**b**) while cell detachment, vacuolations, and cell swelling were observed in heliomycin-treated cells at 1024 µg/mL (**c**) and in combination with AgNPs (AgNPs 1 µg/mL + H 64 µg/mL). (**f**) A mild cell shrinkage was observed in AgNPs-treated cells with MIC (2 µg/mL) (**d**) and in combination with rutin-treated cells (AgNPs 1 µg/mL) + R 64 µg/mL (**e**).

The morphology of EPC cells is one of the significant indicators of cell status. The morphological changes of EPC cells after 24 h exposure to the tested compounds were captured using an inverted microscope. Both the control cells and rutin-treated (1024 µg/mL) cells were large and elongated with good adherence (Figure 8a,b), indicating that the rutin treatment was safe. Most of the heliomycin-treated cells lost their adherence from the culture plate, became spherical and pyknotic, and started to float in the medium, finally leading to their death, indicating a highly cytotoxic effect of heliomycin (Figure 8c). A similar effect was observed to a lower extent for the combination of AgNPs + H (Figure 8f). The AgNPs-treated cells (Figure 8d) and sub-MIC of AgNPs in combination with rutin (Figure 8e) showed extended cytoplasm for intact cells and moderate morphological changes.

#### 2.3.2. Cell Viability Results

**Figure 9:**
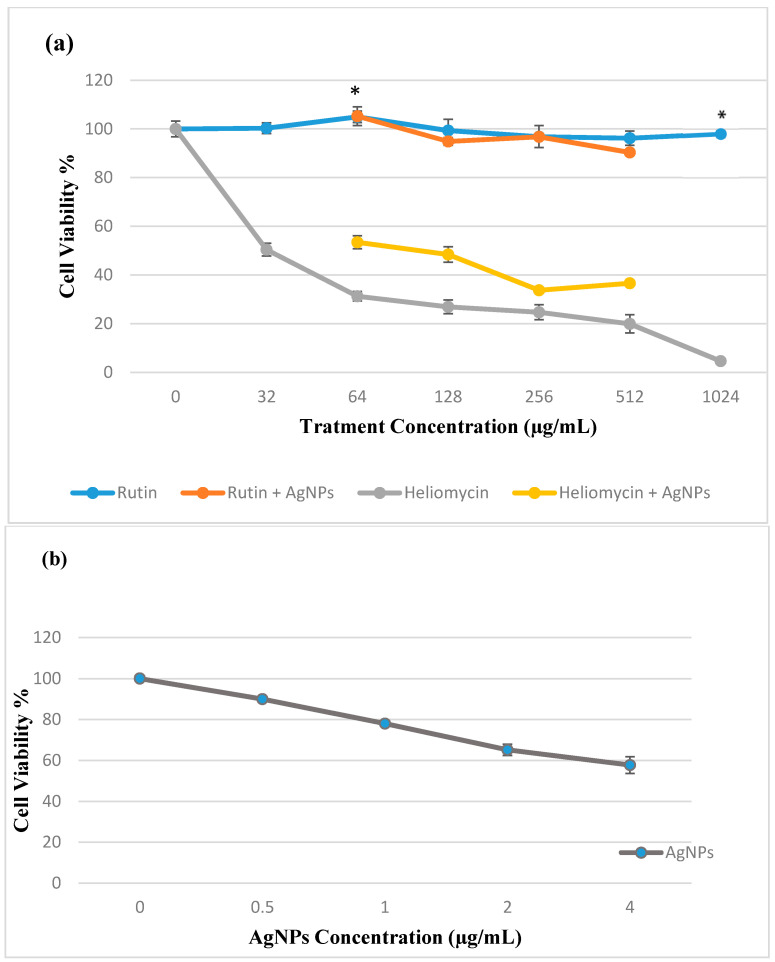
Assessment of in vitro cytotoxicity of tested bioactive compounds on the EPC cell line after 24 h exposure by Alamar blue assay. (**a**) Rutin and heliomycin two-fold serial dilutions sub-MIC combinations with AgNPs (1 µg/mL). (**b**) Cell viability against AgNPs serial dilution. * Statistical significance from control (*p* < 0.05).

Alamar blue assay revealed a high viability of EPC cells (97%) when treated with rutin MIC at 1024 µg/mL with dose-dependent cytotoxicity and with no significant difference (*p* > 0.5) when compared to the negative, untreated control cells. On the other hand, a dose-dependent reduction in cell viability was observed after 24 h incubation with heliomycin. A combination of rutin and AgNPs showed high viability, while a subtherapeutic dose of AgNPs + H showed less cell viability (Figure 9a). AgNPs alone showed moderate viability of 65% at MIC and increased to 78% when subjected to (0.5 MIC) concentration of (1 µg/mL) (Figure 9b). All data are expressed as means ± SD and are represented in a line chart (Figure 9).

## 3. Discussion

The utilization of silver nanoparticles in biomedicine opened doors to several opportunities but has also brought along considerable threats. Some drawbacks associated with AgNPs use have been reported such as treatment accumulation at nontargeted sites, immunogenic and fibrogenic potential, high reactivity, oxidative stress contributing properties, and longer persistence [26]. In addition to the ability to cross various barriers, AgNPs interact with different cellular components such as proteins, lipids, and genetic material [27]. Therefore, a balance between safety and efficacy is necessary for the development of novel nontherapeutic agents.

Bacterial infectious diseases are major threats to cultured fish. However, there is also an increasing need to minimize the use of antibiotics in farming to combat antibiotic resistance [28]. Hence, alternative antimicrobial therapeutics such as metal nanoparticles are widely investigated for their potential medical applications, including their use in fish medicine. Although metal nanoparticles such as AgNPs were reported to show efficient antimicrobial activity [26], limitations for their applications in therapeutic purposes still exist.

The present study examined the synergistic effect of biosynthesized AgNPs with bioactive natural products; from plant origin (rutin), and with a secondary metabolite of microbial origin (heliomycin) on fish pathogens. Improved safety and tolerability were observed by testing on fish cell lines. The green synthesis method was applied to synthesize AgNPs as a safe, eco-friendly, and efficient method that depends on the reduction power of EPS released by *Bacillus nitratireducens*, a soil isolate obtained from the rhizosphere of the wheat plant [29]. The biosynthesized AgNPs exhibited a potent bactericidal effect against *A. hydrophila* with an MBC of 2 µg/mL (Table 1), and an MIC of 2 µg/mL, showing increased values than those previously reported by our group [11], while the MIC of AgNPs against *A. hydrophila* was 16 µg/mL. This high potency of AgNPs could be explained due to their small size of approximately 12 nm and average sample size of 73 nm, good *p* (0.36), and a high zeta potential (−33.8 mV). These results build on the existing evidence [30] in which biosynthesized AgNPs showed a size-dependent effect. Small nanoparticles can pass through pores in the cellular membrane, release a large number of silver ions, generate reactive oxygen species [31], and exhibit a high surface-area-to-volume ratio [32].

Rutin is one of the most abundant flavonoids found in plants. Reports indicate a variety of biological activities, such as analgesic, anti-inflammatory, antiarthritic, and antibacterial effects [33]. Rutin exhibited a dose-dependent response against both tested strains. The MIC values of rutin were observed at 1024 μg/mL against *A. hydrophila* and *P. fluorscens*, which indicate a moderate antibacterial effect. The antibacterial activities of rutin observed in this study were in line with a previous report [34] that reported an MIC of 1100 μg/mL against *A. hydrophila*. Orhan et al. [34] demonstrated rutin’s efficacy against drug-resistant isolates such as *Staphylococcus aureus* and *P. fluorescens aeruginosa* using the broth microdilution method. In addition, the authors found that rutin isolated from tobacco leaves effectively inhibited fungal and bacterial growth. Heliomycin, another tested bioactive compound examined in the current study, is a secondary metabolite produced by actinomycetes AB5 isolated from soil sediment of the river Nile, Egypt [35]. It inhibits the synthesis of ATP and pyrophosphate by uncoupling the processes of respiration and oxidative phosphorylation, which affects the energy metabolism of bacterial cells. Moreover, it inhibits bacterial RNA synthesis [20]. To the best of our knowledge, the effect of heliomycin has not yet been completely studied against fish pathogens. In the present study, heliomycin showed a bacteriostatic effect against *A. hydrophila* and *P. fluorescens* with the MIC at 2048 µg/mL, while the MBC could not be reached for the tested concentrations of the compound. These moderate antibacterial effects of both rutin and heliomycin could be due to their poor solubility in the aqueous phase [33,36]. The addition of biosynthesized AgNPs to rutin (AgNPs + R) and heliomycin (AgNPs + H) improves their bioavailability for a couple of reasons: first, the electrostatic interaction between positive AgNPs and negative electron density on natural phenolic compounds, and second, the larger surface area of AgNPs increases the binding capacity to rutin and heliomycin molecules that potentiate their cellular uptake and hence their overall efficacy against infectious bacteria. This hypothesis is built on the existing evidence about the additive effect of AgNPs with phenolic plant extract [37] in which an AgNPs and curcumin combination gave a better antibacterial effect than the individual treatment. This hypothesis is in accordance with the study conducted by Barbinta-Patrascu et al., who developed hybrid bio-platforms based on a combination of AgNPs and natural compounds [38]. We first examined the enhanced effect of the AgNPs + R and AgNPs + H combination versus individual treatments against *P. fluorescens* using the disc diffusion method (Figure 4), and it resulted in a significant increase in the inhibition zone. These results support our hypothesis, and some reports are in agreement with earlier studies [29,39]. However, the disc diffusion method does not have a parameter to determine the synergistic effect. Therefore, we used the broth microdilution method to perform the synergy study and to identify the break point according to Clinical and Laboratory Standards Institute (CLSI) document VET01-A3 [26]. Data analysis showed an enhanced bactericidal effect of rutin in combination with AgNPs against *A. hydrophila*. This demonstrated an improved percent inhibition of bacterial growth from 40% individual sub-MIC of rutin at 64 µg/mL to approximately 100% combined sub-MIC of AgNPs treatment (Figure 5). The difference in susceptibility to the combined treatment effect of AgNPs + bioactive compound, to be additive or synergistic, depends on the ability of the nanosystem to penetrate the bacterial capsule that formed from the exopolysaccharide biofilm [40], which is still poorly understood due to its spatial and chemical variability and complexity. Hence, in-depth knowledge is required to the nature and composition of the biofilm matrix to properly design the nanosystem that is able to disrupt it [41].

The cytotoxicity of AgNPs, rutin, heliomycin, and their combinations were evaluated on the EPC cell line using Alamar blue assay [42,43]. Measuring the metabolic activity of the mitochondrial enzyme via quantification of its reducing power to convert the oxidized form of resazurin (blue color) to its reduced form (pink color) enables the comparison between the viability of the treated cells and untreated control cells. Low doses of heliomycin were tolerated, but the MIC dose of heliomycin (1024 µg/mL) was highly cytotoxic to EPC as well as its combinations at sub-MIC concentrations with AgNPs (1 µg/mL). These results agree with Abdelfattah et al. [24], in which the high cytotoxic effect of heliomycin against cancer cell lines via inhibiting histone deacetylase enzyme activity was reported. Low doses of AgNPs at MIC (2 µg/mL) exhibited moderate morphological changes and tolerability and are in agreement with previous studies [44,45]. On the other hand, no significant cytotoxicity was observed with the rutin MIC dose (1024 µg/mL, *p* > 0.99), indicating the high safety of the rutin treatment. This could be explained by its reported antioxidant activities [46]. The viability of the EPC cell line was more than 90% without significant change in their morphology after the incubation with rutin MIC and in combination with AgNPs at sub MIC of (1 µg/mL) + R (64 µg/mL) and is in accordance with Barbinta-Patrascu et al. [38], in which the safety and efficacy of the combination of natural molecules with AgNPs were reported to support the synergistic and additive effects of these treatments and open a possibility for both biomedical and aquaculture applications.

## 4. Material and Methods

### 4.1. Extraction and Characterization of Rutin

The natural flavonoid rutin was extracted from the dried ground leaves of *Ruta graveneoles*, as previously described [47]. Briefly, the dried leaves (1000 g) were mixed with 70% ethanol at room temperature and then filtered, extraction was repeated five times, and was followed by evaporation under reduced pressure. The aqueous layer was collected and left to stand for three days. A yellow precipitate separated out of the solution. The precipitate was filtrated and washed three times with dichloromethane: ethyl acetate (2:1) to give 500 mg of a yellow solid compound. Rutin was obtained from the library of the Natural Product Lab., Faculty of Science, Helwan University, Cairo, Egypt.

### 4.2. Extraction and Characterization of Heliomycin

Heliomycin was extracted from the culture broth of actinomycete AB5, as described previously [35]. In brief, the fermentation broth was harvested after four days and centrifuged at 4000 rpm for 10 min. The resulting mycelial cake was extracted three times with methanol, and the aqueous phase was extracted with ethyl acetate. A formed brown crude extract was dissolved in methanol and left to stand overnight. A yellow precipitate was split apart, filtrated, and washed three times with methanol to give a yellow solid compound. The structure of the extracted compound was characterized by spectroscopic methods such as nuclear magnetic resonance (NMR) and mass spectrometry. Heliomycin was obtained from the library of the Marine Natural Product Unit, Natural Product Lab., Faculty of Science, Helwan University, Cairo, Egypt.

### 4.3. Biosynthesis and Characterization of Silver Nanoparticles (AgNPs)

AgNPs were synthesized by the reduction of AgNO_3_ using EPS released by *Bacillus nitratireducens* isolated from soil, according to Wenjie Jian et al. [35]. Characterization of particle size and zeta potential was performed based on dynamic light scattering (DLS) by using a Malvern Zetasizer Nano ZS device. This characterization analysis was performed in triplicate on diluted AgNPs suspension prepared in de-ionized distilled water. The DLS was measured at a 90° scattering angle at 25 °C.

### 4.4. Assessment of the Antibacterial Activities of AgNPs and Natural Phenolic Compounds

#### 4.4.1. Bacterial Strains and Growth Conditions

Aliquots of two fish pathogenic bacterial isolates *A. hydrophila* (252/13) and *P. fluorescens* were obtained from the Micro Bank in the Clinical Division of Fish Medicine, University of Veterinary Medicine, Vienna, Austria. Confirmatory identification was performed using MALDI-TOF (Bruker, Karlsruhe, Germany) and by analytical profile index (API). Both the bacterial isolates were confirmed using 16S rRNA sequencing, as previously reported [13]. Then, under aseptic conditions, a loop from each pure strain was streaked on Müller–Hinton (MH) agar plates (Sigma-Aldrich, Vienna, Austria) and incubated at 22 °C for 24 h.

#### 4.4.2. Bacterial Growth Inhibition Assay

A single colony of each isolate was inoculated in 10 mL Müller–Hinton (MH) broth and incubated for 24 h in a shaker incubator at 126 rpm at 22 °C [9]. The bacterial growth was monitored at optical density (OD) 600 nm using a spectrophotometer (Eppendorf BioPhotometer, Eppendorf, Hamburg, Germany) to get 10^6^ colony forming units (CFU/mL) cells. Equal volumes of each bacterial strain were mixed with tested compounds to reach a final concentration of 5 × 10^5^ CFU/mL. A negative control was prepared by mixing equal volumes of bacteria and MH media.

#### 4.4.3. Minimum Inhibitory Concentration (MIC) Assay

The MIC assay for AgNPs, rutin, heliomycin, AgNPs + R, and AgNPs + H against multiple drug-resistant strains of *A. hydrophila* and *P. fluorescens* was performed by the standard broth microdilution method according to the Clinical and Laboratory Standards Institute (CLSI) [48]. All experimental analyses were performed in triplicate. The bacterial growth was evaluated visually, and the MIC was determined as the lowest concentration that completely inhibited the growth.

Minimum Bactericidal Concentration (MBC) Assay:

To evaluate the MBC, 100 μL was taken out from the wells without visible cell growth and incubated on agar plates, followed by incubation at 22 °C for 24 h. After incubation, CFUs were counted and digital images of the plates were captured. The MBC was determined as the lowest concentration that inhibited colony formation. The assay was performed in triplicate, and the results, repeated twice or more, were considered [49].

#### 4.4.4. Synergy Study

We examined different preparations of sub-MICs for AgNPs in combination with rutin (AgNPs + R) and heliomycin (AgNPs + H), as described before [34]. We selected rutin and heliomycin concentrations at 512 μg/mL (0.5 MIC), 256 μg/mL (0.25 MIC), 128 μg/mL (0.125 MIC), and 64 μg/mL (0.062 MIC) that could be used synergistically with AgNPs sub-MIC of 1 μg/mL (0.5 MIC) (Table 2). The fractional inhibitory concentration index (FICI) values were calculated as follows:

FICI = (MIC of AgNPs in combination/MIC of AgNPs alone) + (MIC of rutin in combination/MIC of rutin alone)(1)

FICI = (MIC of AgNPs in combination/MIC of AgNPs alone) + (MIC of heliomycin in combination/MIC of heliomycin alone)(2)

The FICI values for synergism, indifference, and antagonism were deduced as ≤0.5, >0.5 to ≤4.0, and >4.0, respectively.

#### 4.4.5. Disc Diffusion Method

The possible synergistic/additive antimicrobial effects of AgNPs in combination with rutin (AgNPs + R) and heliomycin (AgNPs + H) against *P. fluorescens* was visualized and tested using the disc diffusion method, according to CLSI guidelines [50].

#### 4.4.6. UV–Visible Absorption

To confirm the bacterial growth inhibition, the OD at 600 nm of the inoculated broth media of all the replicate test tubes corresponding to the sensitive isolates during the MIC assay was measured using a 96-well plate absorbance reader, Tecan Sunrise (Männdorf, Switzerland) [51]. The low absorbance capacity indicated less bacterial growth and vice versa. Blanks used for the OD measurements were the same as the MIC blank controls. The antibacterial effect of each dose for bioactive compounds was expressed as percentage inhibition (%) of the bacterial growth according to the following equation [52]:

(3) $$\mathrm{Inhibition}\;\left(\%\right)=1-\left(\frac{\mathrm{OD}\;\mathrm{sample}}{\mathrm{OD}\;\mathrm{control}}\right)\times 100$$

The growth inhibition percent of each replicate was calculated from the obtained OD_600_ measurements, and the results were expressed as mean percentages ± standard deviation (SD) in histograms comparing the species-specific antibacterial efficacy of the compound preparations.

### 4.5. Cytotoxicity Assay

#### 4.5.1. Fish Cell Lines and Culture Condition

The fish cell line EPC, from carp (*Cyprinus carpio*), was cultivated on a 24-well tissue culture plate containing minimal essential medium (MEM, Gibco^®^, New York, NY, USA), supplemented with 2% fetal bovine serum. EPC cells were incubated at 20 °C for 24 h until a confluent monolayer and an approximate cell seeding density of 2 × 10^5^ was achieved.

#### 4.5.2. Cell Morphology Examination

The culture medium was replaced with fresh medium containing selected treatment concentrations of bioactive compounds according to the MIC data given in Table 1 and Table 2. After incubation at 19 °C for 24 h, EPC cells morphology was recorded using an inverted phase-contrast microscope (Nikon, Tokyo, Japan) at 20× magnification.

#### 4.5.3. Cytotoxicity and Cell Viability Assessment

In vitro safety assessment of bioactive compounds was performed [53] as follows. Briefly, 200 µL MEM containing approximately 4 × 10^4^ EPC cells was seeded into each well of 96-well plates and allowed to adhere at a temperature of 19 °C. After the formation of the confluent monolayer, all media were replaced with the new media containing a two-fold serial dilution of the bioactive compounds rutin (0–1024 µg/mL), heliomycin (0−1024 µg/mL), AgNPs (0–4 µg/mL), and sub-MIC combinations of AgNPs + R and AgNPs + H (Table 2). The negative control, growth control, and media blank were included, and then the plate was incubated for 24 h at 19 °C. The medium was then removed, and the wells were washed with phosphate buffer saline (PBS) twice to remove any residual particles. The viability of EPC cells was tested using an Alamar blue high-sensitivity assay (Invitrogen^®^, Oregon, OR, USA) according to the manufacturer’s instructions [54,55]. After 3 h incubation, the wells were read using a spectrophotometric plate reader, Tecan Sunrise (Männdorf, Switzerland), at respective oxidized and reduced forms at wavelengths of 550 nm and 600 nm. The percentage of surviving cells was calculated for each well, and the survival percent (viability) was expressed as mean percent ± SD. The assay was performed in triplicate for each treatment and single control well per assay.

### 4.6. Statistical Analysis

All data were expressed as means ± SD obtained from ≥3 independent experiments performed on different days with at least one replicate per experiment. The obtained data were checked using Levene’s test. The statistical analysis was performed using SPSS 25 (IBM) software for the covariance (ANOVA) [56] with *post hoc* Dunnett’s T3 test for paired comparison of the means. The statistical significance of differences from control values was taken at *p* < 0.05, evaluating the data at a >95% confidence level. All *p*-values of <0.01 and <0.001 were considered significant and highly significant, respectively.

## 5. Conclusions

Biosynthesized AgNPs, rutin, and heliomycin demonstrated in vitro dose-dependent antimicrobial efficacy against *A. hydrophila* and *P. fluorescens*. The enhanced effect of rutin and heliomycin was achieved by a combination of their subtherapeutic doses with AgNPs, wherein AgNPs could improve their solubility and hence potency. In terms of safety among the tested compounds when tested on EPC cells, rutin exhibited high tolerability, followed by the sub-MIC combination of (AgNPs + rutin). Hence, rutin as a natural flavonoid compound is an effective and safe antibacterial agent and could be used in combination with AgNPs for disease management in aquaculture. Further in vivo studies are required to investigate the antimicrobial efficacy and safety of these combinations on living fish.

## Figures and Tables

**Table 1:** Identified minimum inhibitory concentration (MIC) and minimum bactericidal concentration (MBC) values of rutin (R), heliomycin (H), and silver nanoparticles (AgNPs) against *A*. *hydrophila* and *P. fluorescens*.

Compound	Strain	MIC (μg/mL)	MBC (μg/mL)
Rutin	*A. hydrophila*	1024	2048
	*P. fluorescens*	1024	2048
Heliomycin	*A. hydrophila*	2048	(-)
	*P. fluorescens*	1024	(-)
AgNPs	*A. hydrophila*	2	20
	*P. fluorescens*	4	20

(-): MIC and MBC values that did not reach for all doses examined in this study.

**Table 2:** Selected combinations of sub-MIC doses for synergy study.

Compound	Strain	Selected Sub-MIC (μg/mL)	Concentrations Used in Synergy Study (μg/mL)
Rutin	-*A hydrophila.* -*P. fluorescens*	R512, R256, R128, R64	R 512 (μg/mL) + AgNPs 1 µg/mL R 256 (μg/mL) + AgNPs 1 µg/mL R 128 (μg/mL) + AgNPs 1 µg/mL R 64 (μg/mL) + AgNPs 1 µg/mL
Heliomycin	-*A. hydrophila* -*P. fluorescens*	H512, H256, H128, H64	H 512 (μg/mL) + AgNPs 1 µg/mL H 256 (μg/mL) + AgNPs 1 µg/mL H 128 (μg/mL) + AgNPs 1 µg/mL H 64 (μg/mL) + AgNPs 1 µg/mL
AgNPs	-*A. hydrophila* -*P. fluorescens*	1	

**Table 3:** Results of interaction study between rutin (R) and silver nanoparticles (AgNPs).

Strain	MIC_AgNPs_/MIC _R_ (Alone, µg/mL)	MIC_AgNPs_/MIC _R_ (in Combination, µg/mL)	FICI	Effect
*A. hydrophila*	2/1024	1/64	0.562	A
*P. fluorescens*	4/1024	1/64	0.3125	S

FICI, fractional inhibitory concentration index; S, synergy; A, additivity. The FICI interpretation: ≤0.5, synergy; 0.5–4.0, indifference; and >4.0, antagonism.

**Table 4:** Results of interaction study between heliomycin (H) + silver nanoparticles (AgNPs).

Strain	MIC_AgNPs_/MIC _H_ (Alone, µg/mL)	MIC_AgNPs_/MIC _H_ (in Combination, µg/mL)	FICI	
*A. hydrophila*	2/2048	1/64	0.531	A
*P. fluorescens*	4/1024	1/64	0.3125	S

FICI, fractional inhibitory concentration index; S, synergy; A, additivity. The FIC indices were interpreted as follows: ≤0.5, synergy; 0.5–4.0, indifference; and >4.0, antagonism.

**Table 5:** Zone of inhibition of compounds against *P. fluorescens.*

Bioactive Compound	Strain	Zone of Inhibition
Rutin (512 µg/mL)	*P. fluorescens*	9 ± 0.2 mm ^a^
Heliomycin (512 µg/mL)	*P. fluorescens*	8 ± 0.3 mm ^a^
AgNPs (2 µg/mL)	*P. fluorescens*	8 ± 0.1 mm ^a^
AgNPs (2 µg/mL) + Rutin (512 µg/mL)	*P. fluorescens*	23 ± 0.8 mm ^b^
AgNPs (2 µg/mL) + Heliomycin (512 µg/mL)	*P. fluorescens*	22 ± 0.5 mm ^b^
Tetracycline (Positive Control)	*P. fluorescens*	30 ± 0.1 mm ^c^

The values are expressed as means ± SD. Different letters (a, b and c) in the same column mean that they are significantly different (*p* ≤ 0.05).

## Data Availability

Please refer to suggested Data Availability Statements in section “MDPI Research Data Policies” at https://www.mdpi.com/ethics.
